# Patterns of cannabis use, perception of harm, and perceived impact of legislative change in an online sample of young adults from Lebanon: insight on recreational users versus dual motive users

**DOI:** 10.1186/s12954-024-00958-3

**Published:** 2024-02-15

**Authors:** Lilian Ghandour, Andre Slim, Nada Abbas, Joseph El-Khoury

**Affiliations:** 1 Department of Epidemiology and Population Health, Faculty of Health Sciences, P.O. Box 11-0236, Beirut, Lebanon; 2 Department of Psychiatry, The Valens Clinic, Business Bay, Dubai, United Arab Emirates

**Keywords:** Cannabis, Substance abuse, Drugs, Lebanon, Adolescents, Medical cannabis, Recreational cannabis

## Abstract

**Background:**

Lebanon remains as one of the major sources of cannabis worldwide. In 2020, its government passed a legislation enabling the cultivation of local medicinal cannabis. This first study following the legislative change examines the overlapping use of cannabis for recreational/medicinal purposes and characteristics of the distinct cannabis user types.

**Methods:**

A total of 1230 young adults (18–24 years) filled an anonymous online survey in early 2020.

**Results:**

Young adults in the sample were distributed as follows: 33% 18–20 years; 60% males; 94% Lebanese; 75% students; and 89% living with family. The older young adults (21–24), males, those employed, living with non-family members, and who perceived themselves as being a little/lot richer than most were statistically significantly more present in the cannabis user subtypes (recreational only or recreational/medicinal) than non-cannabis users. When dual recreational/medicinal users are compared to recreational users only, the latter seemed to have a more conservative profile of behaviours, attitudes, and perceptions and acts of harm. The prevalence ratio comparing the prevalence of users supporting consuming cannabis “once or twice” in dual motive users vs. recreational users only was 1.13 for “once or twice”, 1.25 for “occasionally”, 1.64 for “regularly”, and 2.4 for “daily”. Any other illicit drug use was reported by 1% of the non-cannabis users, 36% of the recreational users only, and 58% of the recreational/medicinal users (*p-*value < 0.01). Similarly, any prescription drug use was reported by 3% of the non-cannabis users, 16% of the recreational users only, and 28% of both recreational/medicinal users (*p-*value < 0.01).

**Conclusion:**

The interface between recreational and medicinal cannabis use is complex. Dual motive users may warrant special attention as a subpopulation of cannabis users. This is relevant to contexts experiencing medicinal cannabis legislation changes, such as Lebanon, as policymakers and implementers should be sensitized to the emerging evidence for more data-informed policy changes.

**Supplementary Information:**

The online version contains supplementary material available at 10.1186/s12954-024-00958-3.

## Introduction

Globally, more than 40 countries have legalized cannabis fully or partially for medicinal use, which appears to have increased daily use of cannabis and its health-related impacts in some parts of the world [1]. In Canada, the prevalence of recreational cannabis use has increased by 26% since its legalization for medical purposes in 1999 [2]. Generally, though the evidence remains inconclusive with some studies having reported “post-legalization” increases in recreational cannabis use while others have found no changes [3].

As more countries move towards the legalization of cannabis for medical use, it is important to understand similarities and differences between medical, recreational, and dual motive users.

Existing comparisons is primarily conducted in large-scale population studies from the US and Canada, and focused on comparing medical vs. recreational users, showing that medical cannabis users (vs. recreational users) have a worse health status and engage in more daily cannabis use [4]. A lesser number of studies have focused on understanding the profile of dual motive users, who seem to report more daily cannabis use and more alcohol and tobacco use compared to medical-only users [5]. The interest in the application of the harm reduction approach to cannabis precedes the liberalization of drug laws on its use. It has been proposed that cannabis itself has acted as a substitute to Class A drugs, including for opiates and stimulants [6, 7]. Exploring how the public uses cannabis and for what purpose has become more relevant now that research on the substance has become more accessible [8]. Some studies have confirmed that knowledge on the benefits and harm of cannabis among users remains subjective while there is evidence that placing social boundaries around cannabis use has prevented abuse, especially in older users [9, 10].

Lebanon remains as one of the major sources of cannabis worldwide, both for export and local consumption [11]. This small country (10,452 km^2^) in the eastern Mediterranean region is the second most cannabis producing country in near and Middle East/South-West Asia, following Afghanistan [1, 12, 13]. In 2020, the Lebanese government passed legislation enabling the cultivation of local medicinal cannabis (< 1% tetrahydrocannabinol) [14], which neither describes the process by which the medicinal cannabis would be made available to the public in Lebanon, nor does it address the legal status of recreational cannabis [14]. Meanwhile, cannabis remains largely demonized with heavy sentences served to anyone suspected of using or selling or cultivating cannabis [14]. Even socially, cannabis users are described derogatorily in Arabic as “Hashash” (consumer of Hashish).

Published evidence has so far documented clear and complex differences across cannabis user types. This present paper aims to add to the scientific literature exploring similarities and differences across cannabis use types, namely focusing on the less studied distinction between recreational users only and dual motive users in the patterns of cannabis and other substance use, perceived cannabis harms, attitudes towards legalization and its perceived impact on cannabis, and other substance use. The study findings will also describe and document the landscape of cannabis consumption before legislative changes become effective, necessary for guiding public health policies and apply preemptive harm reduction policies [15].

## Methods

### Sample recruitment and data collection

Following the approval of the American University of Beirut Institutional Review Board (IRB) in November 2020 [protocol # SBS-2020-0421], an online survey was launched in January 2021 administered via LimeSurvey. Since cannabis use in Lebanon is illegal, an anonymous online survey was the most appropriate approach. It is worth noting that the data period also coincided with the COVID-19 pandemic lockdown further necessitating an online convenience sample approach. Questions pertaining to cannabis use within the last 12 months (i.e. 2020) might be impacted by COVID-19, the lockdown, and the consequences thereof. However, no evidence has yet corroborated this interplay.

Young adults living in Lebanon and aged 18–24 were invited to participate in the study. The team circulated the invitation text and the link to the survey via various social media platforms (personal and professional accounts on Facebook, Instagram, and Twitter); the link was also circulated via WhatsApp among personal contacts/groups. The invitation text included important information including study purpose, inclusion criteria, and other relevant information regarding anonymity and data usage for maximum transparency. Once the link was clicked, respondents were taken to an online consent form page, where they were asked to read carefully and click submit [i.e. consent] if they wish to participate. The final sample by March 2021 included 1230 young adults who had consented and completed the online self-administered questionnaire.

### Data collection form

The survey was developed in English by the investigators, building upon the previous surveys [16], and incorporating additional questions relevant to the Lebanese context. The questionnaire was available in both English and Arabic for the participants to complete in their language of preference. The survey included questions focused on: sociodemographics, patterns of consumption of cannabis, attitudes towards the use and legalization of cannabis (recreational and medical), perception of cannabis harms, and consumption of other drugs.

### Outcome measure

We asked respondents to report whether they have ever used cannabis for medical purposes [defined in the questionnaire as “any consumption to cure, treat, or prevent a physical or psychiatric disease”] or recreational purposes [defined as “any consumption for pleasure or to induce an alternate state of consciousness”]. A separate question inquired whether the “medicinal use” has been authorized or prescribed by a medical practitioner. We focused on lifetime use in this paper and the overlapping use of cannabis for medical and recreational use. In this sample, 436 young adults (35%) “Preferred not to answer (PNA)” the question on “lifetime recreational cannabis use”, and seven young adults only reported using cannabis for medicinal purposes only. In order to allow for comparisons across user types, we chose to exclude the seven medicinal users only from the analyses and include those who did not disclose recreational use as PNA recreational/non-medicinal user as a separate category of interest. Thus, for this paper, our main outcome measure was cannabis user type: *non-cannabis user* [responded negatively to both recreational/medicinal cannabis use questions], *recreational users only* [responded negatively to medicinal use and positively to recreational use], *recreational/medicinal users* [responded positively to both questions], and *PNA recreational/non-medicinal users* [young adults with unknown recreational status and confirmed non-medicinal use].

### Data analysis

Data were analysed using Stata version 14. Pearson’s Chi-square was run to examine associations between categorical variables.

## Results

### Sociodemographic profile of the online young adult sample

Table 1 describes the sociodemographic characteristics of the online young adult sample. A third of the respondents (32.36%, *n* = 398) were 18–20 years of age, although the majority (67.64%, *n* = 832) were aged 21–24 years old. The sample comprised of slightly more males (59.6%, *n* = 723) and was predominantly Lebanese and/or Lebanese with dual citizenship (94.39%, *n* = 1161) and residing in Lebanon (93%, *n* = 1123) for most of their lives. Around 75% were students at the time of the online survey, with 55.74% (*n* = 665) having completed at least a bachelor’s degree. A third (32.2%, *n* = 384) were employed. The vast majority (89.27%, *n* = 1082) were living with family (e.g. parents or guardians or spouse or siblings). When asked to compare themselves to other people their age, 14.9% (*n* = 176) self-reported being poorer or a lot poorer, and 32.6% (*n* = 385) being richer or a lot richer.

**Table 1:** Sociodemographic characteristics of the 1230 young adult online survey respondents

	Valid % (*n*)
Age (in years)	
18–20	32.36 (398)
21–24	67.64 (832)
Gender	
Female	40.4 (490)
Male	59.6 (723)
Nationality	
Lebanese	98.39 (1161)
Non-Lebanese	1.61 (19)
Country of residence for most of young adult life	
Lebanon	93 (1123)
Other	7 (85)
Current student status	
Not a student	24.4 (293)
Student full-/part-time	75.6 (908)
Highest level of education	
Secondary school/high school	22.3 (266)
Some university no degree	21.96 (262)
Bachelor’s degree	44.26 (528)
Master’s/MD PhD	11.48 (137)
Current job status	
Working	32.2 (384)
Not working	67.8 (810)
Living arrangement	
With family member	89.27(1082)
Others (including alone, friends, and roommates)	10.73 (130)
Perceived SES	
Little/LOT poorer than most	14.9 (176)
About the same than most	52.5 (621)
Little/LOT richer than most	32.6 (385)

### Cannabis use for recreational and/or medicinal purposes

Out of 1230 young adults, only 8.5% (*n* = 105) reported never trying cannabis for recreational purposes although about one-third of the sample (35.5%, *n* = 436) “preferred not to answer” this question; 56% (*n* = 689) of the sample reported lifetime recreational cannabis use (Table 2). Of those, approximately 80% (*n* = 546) were past-year users (i.e. any use of cannabis for recreational purposes within the past year); 22.2% reported trying it for the first time (i.e. initiated recreational cannabis use) within the past year (Table 2). In terms of frequency of use, about half of the past-year users (*n* = 546) reported using cannabis at least 1–2 times per week (Table 2).

**Table 2:** Patterns of cannabis use in 1230 young adult online respondents

Lifetime recreational cannabis use (n = 1230)	% (*n*)
Yes	56.0 (689)
No	8.5 (105)
Prefer not to answer	35.5 (436)
First time used cannabis recreationally in lifetime recreational users (*n* = 674)	
Within the past month	7.4 (50)
More than a month ago but within the past 6 months	6.7 (45)
More than 6 months ago but less than a year ago	8.6 (58)
More than a year ago	77.3 (521)
Last time used cannabis recreationally in lifetime recreational users (*n* = 640)	
Within the past month	59.1 (378)
More than a month ago but within the past 6 months	18.3 (117)
More than 6 months ago but less than a year ago	7.9 (51)
More than a year ago	14.7 (94)
Frequency of recreational cannabis use in past-year recreational users (*n* = 520)	
Less than once per week/month	45.2 (235)
1–2 times per week or more	54.8 (285)
Lifetime medicinal cannabis use (*n* = 1169)	
Yes, as per healthcare provider/pharmacist advice	2.1 (25)
Yes, without any physician’s advice	14.4 (168)
No, never tried cannabis for medicinal purposes	83.5 (976)
First time used cannabis for medicinal purposes among lifetime users (*n* = 182)	
Within the past month	18.7 (34)
More than a month ago but within the past 6 months	15.9 (29)
More than 6 months ago but less than a year ago	10.9 (20)
More than a year ago	54.5 (99)
Last time used cannabis for medicinal purposes among lifetime users (*n* = 173)	
Within the past month	49.7 (86)
More than a month ago but within the past 6 months	17.9 (31)
More than 6 months ago but less than a year ago	9.2 (16)
More than a year ago	23.2 (40)
Frequency of medicinal cannabis use among past-year medicinal users (*n* = 133)	
Less than once per week/month	48.1 (64)
1–2 times per week or more	51.9 (69)
Main one reason reported reason for trying cannabis (*n* = 674)	
To experiment (to see what it’s like)	67.22 (453)
To relax or relieve tension	10.53 (71)
To get high	8.75 (59)
Because it is safer than any other illegal drug	3.12 (21)
To help with my negative feelings/emotions	2.82 (19)
Because of anger or frustration	2.52 (17)
To get some sleep	1.93 (13)
To avoid dealing with my problems or troubles	1.48 (10)
To relieve physical pain	1.04 (7)
Because I was not getting the effect, I needed from another drug	0.29 (2)
To get through the day	0.15 (1)
As a substitute for heroin	0.15 (1)
Company first time tried cannabis: (multiple options allowed) (*n* = 693)	
Alone	6.49 (45)
Close friend	77.92 (540)
Boyfriend/girlfriend	6.64 (46)
Siblings	4.04 (28)
Spouse	0.29 (2)
Co-worker	1.73 (12)
Other	2.89 (20)
Ever smoked the dried plant in a rolled cigarette (joint), pipe, or bong (*n* = 624)	
No	15.06 (94)
Yes	84.94 (530)
Ever smoked the liquid or wax marijuana in an electronic cigarette, also known as vaping (*n* = 598)	
No	60.7 (363)
Yes	39.3 (235)
Ever eaten baked goods or candies containing marijuana products (*n* = 616)	
No	34.25 (211)
Yes	65.75 (405)
Ever drank beverages containing marijuana products (*n* = 600)	
No	74.67 (448)
Yes	25.33 (152)
Ever used oils and tinctures that can be applied to the skin (*n* = 601)	
No	71.55 (430)
Yes	28.45 (171)

Of the 1230 young adults, 15.7% (*n* = 193) reported lifetime medicinal cannabis use [defined as “any consumption to cure, treat, or prevent a physical or psychiatric disease”]. Only 2% of the total sample was “prescribed” cannabis by a healthcare provider/pharmacist, and the remainder (13.7%) took it without any physician’s advice (Table 2). Of the lifetime medicinal cannabis users, about 70% used cannabis medicinally within the preceding year (52% of whom used it at least once or twice per week). In terms of onset of use, 43% had initiated medicinal cannabis use within the past year (Table 2).

We also examined the overlapping use of cannabis for medical and recreational use. Excluding the seven medicinal only users (who reported never using cannabis recreationally), the remainder of the sample (*n* = 1223) was distributed as follows: 8.3% (*n* = 102) were never cannabis users, 41% (*n* = 503) were recreational only users, 15% (*n* = 186) were both recreational/medicinal users, and 35% (*n* = 432) were PNA recreational/non-medicinal users.

We tried to understand transitions between recreational and medicinal use by examining both ages of onset. Of the 186 young adult cannabis recreational/medicinal users, all had used cannabis recreationally first [mean difference: 2.14 years SD: 2.13], and about 27% reported using cannabis for recreational and medicinal reasons within the same year.

When asked about the *one main* reason for trying cannabis, two-thirds (67.22%, *n* = 453) of the 689 cannabis users (recreational and/or medicinal) said *“to experiment and see what it is like”*, followed by 10.53% (*n* = 71) who used cannabis *“to relax or relieve tension”*, and then by 8.75% (*n* = 59) who admitted to using cannabis “*to get high*”. The majority (77.92%, *n* = 540) were in the company of a close friend when they first tried cannabis (Table 2).

Cannabis consumption methods varied (Table 2): 85% smoked cannabis in a rolled cigarette, pipe, or bong; 39% smoked the liquid or wax cannabis in an electronic cigarette; 66% ate baked goods or candies containing cannabis products; 25% had beverages containing cannabis; and 28% used oils and tinctures that can be applied on the skin.

### Comparing cannabis user types

The next paragraphs describe differences between cannabis user types, by sociodemographics (Table 3), other substance use (Table 4), attitudes towards cannabis use for recreational and medicinal purposes (Table 5), perceived harmfulness of cannabis (Table 6), and attitudes towards cannabis legalization and the newly passed Lebanese law (Table 7).

**Table 3:** Sociodemographics of the cannabis user types (*n* = 1223)

	Total (*n* = 1223)		Neither recreational nor medicinal (*N* = 102)	Only recreational (*N* = 503)		Both recreational and medicinal (*N* = 186)		PNA^a^ recreational, no medicinal (*N* = 432)
Age (in years) **	% (*n*)		% (*n*)	% (*n*)		% (*n*)		% (*n*)
18–20	32.38 (396)		40.20 (41)	29.82 (150)		19.89 (37)		38.89 (168)
21–24	67.62 (827)		59.8 (61)	70.18 (353)		80.11 (149)		61.11 (264)
Gender **								
Female	39.98 (489)		49.02 (50)	29.23 (147)		24.73 (46)		56.94 (246)
Male	58.71 (718)		50.98 (52)	68.79 (346)		74.19 (138)		42.13 (182)
Prefer not to answer^b^	1.31 (16)		0 (0)	1.98 (10)		1.08 (2)		0.93 (4)
Nationality *								
Lebanese	98.38 (1154)		94.95 (94)	98.76 (479)		96.68 (175)		99.51 (406)
Non-Lebanese	1.62 (19)		5.05 (5)	1.24 (6)		3.32 (6)		0.49 (2)
Country of residence for most of young adult life								
Lebanon	98.23 (1110)		98.98 (97)	98.07 (457)		97.63 (165)		98.49 (391)
Other	1.77 (20)		1.02 (1)	1.93 (9)		2.37 (4)		1.51 (6)
Current student status**								
Not a student	24.12 (288)		17.17 (17)	31.25 (155)		38.07(67)		11.58 (49)
Student full-/part-time	75.88 (906)		82.83 (82)	68.75 (332)		61.93 (109)		88.42 (374)
Highest level of education								
Secondary/high school	21.94 (256)		26.26 (26)	20.54 (99)		17.05 (30)		24.63 (101)
Some university no degree	22.28 (260)		21.21 (21)	20.33 (98)		27.27 (48)		22.68 (93)
Bachelor’s degree	44.64 (521)		40.40 (40)	46.68 (225)		44.89 (79)		43.17 (177)
Master’s/MD PhD	11.14 (130)		12.13 (12)	12.45 (60)		10.7 (19)		9.52 (39)
Current job status **								
Working	32.1 (381)		27.55 (27)	37.2 (183)		46.93 (84)		20.81 (87)
Not working	67.9 (806)		72.45 (71)	62.8 (309)		53.07 (95)		79.19 (331)
Living arrangement **								
With family member		89.13 (1074)	92 (92)		86.9 (431)		80.87 (148)	94.6 (403)
Others (including alone, friends, and roommates)		10.87 (131)	8 (8)		13.1 (65)		19.13 (35)	5.4 (23)
Perceived SES **								
Little/LOT poorer than most		14.97 (176)	21 (21)		15.07 (74)		21.67 (39)	10.37 (42)
About the same than most		52.55 (618)	55 (55)		47.86 (235)		46.67 (84)	60.25 (244)
Little/LOT richer than most		32.48 (382)	24 (24)		37.06 (182)		31.66 (57)	29.38 (119)

^a^Prefer not to answer

^b^Includes 10 young adults who preferred not to identify as neither “male” nor “female”

* means *p*-value < 0.05 and ** means *p*-value < 0.01

**Table 4:** Other substance use and cannabis user types (n = 1223)

	Total (*n* = 1223)	Neither recreational nor medicinal (*N* = 102)	Only recreational (*N* = 503)	Both recreational and medicinal (*N* = 186)	PNA recreational, no medicinal (*N* = 432)
	% (*n*)	% (*n*)	% (*n*)	% (*n*)	% (*n*)
Cocaine use	10.55 (129)	0 (0)	14.52 (73)	29.57 (55)	0.23 (1)
Crack/freebase use	1.8 (22)	0 (0)	2.38 (12)	5.37 (10)	0 (0)
Stimulants use^a^	7.69 (94)	0 (0)	10.74 (54)	20.43 (38)	0.46 (2)
Heroin use	1.15 (14)	0 (0)	1 (5)	4.84 (9)	0 (0)
Hallucinogens use^b^	9.57 (117)	0 (0)	12.13 (61)	28.52 (53)	0.69 (3)
Ecstasy/MDMA use	11.69 (143)	0 (0)	16.7 (84)	31.72 (59)	0 (0)
GHB use	0.82 (10)	0 (0)	0.8 (4)	3.23 (6)	0 (0)
Salvia/sylvia/spice use	11.94 (146)	0.98 (1)	17.49 (88)	28.49 (53)	0.92 (4)
Any illicit drug other than cannabis **	**24.6 (280)**	**1.05 (1)**	**36.17 (174)**	**57.56 (99)**	**1.54 (6)**
Benzodiazepines use^c^	10.47 (128)	2.94 (3)	13.12 (66)	23.12 (43)	3.7 (16)
Other opiates use^d^	6.3 (77)	0 (0)	8.75 (44)	13.97 (26)	1.62 (7)
Any prescription drug use**	**13.31 (154)**	**3.12 (3)**	**16.36 (80)**	**28.25 (50)**	**5.32 (21)**
Alcohol use **	78.99 (966)	53.92 (55)	95.43 (480)	91.94 (171)	60.19 (260)

The numbers are in bold to highlight the interplay between cannabis use and other illicit/prescription drug use

^**^ *P*-value < 0.01

^a^Speed, Ice, Crystal Meth, Captagon, and Ritalin

^b^LSD, Acid, Mushrooms, and Ketamine

^c^Xanax, Rivotril, Ativan, Lexotanil, and Valium

^d^Tramal, CEMO, and Algocod

**Table 5:** Attitudes towards cannabis use by cannabis user types (N = 1223)

	Total (*n* = 1223)	Neither recreational nor medicinal (*N* = 102)	Only recreational (N = 503)	Both recreational and medicinal (*N* = 186)	PNA recreational, no medicinal (*N* = 432)
	% (*n*)	% (*n*)	% (*n*)	% (*n*)	% (*n*)
Attitudes towards cannabis use for recreational purposes					
If only used once or twice **					
Neutral/reserved/opposing	57.32 (701)	71.57 (73)	46.13 (232)	38.71 (72)	75 (324)
Encouraging/supportive	**36.88 (451)**	**24.51 (25)**	**50.89 (256)**	**58.06 (108)**	**14.35 (62)**
PNA/do not know	5.8 (71)	3.92 (4)	2.98 (15)	3.23 (6)	10.65 (46)
If used once in a while or occasionally **					
Neutral/reserved/opposing	48.08 (588)	64.71 (66)	35.98 (181)	16.67 (31)	71.76 (310)
Encouraging/supportive	**45.54 (557)**	**31.37 (32)**	**60.83 (306)**	**79.03 (147)**	**16.67 (72)**
PNA/do not know	6.38 (78)	3.92 (4)	3.18 (16)	4.3 (8)	11.57 (50)
If used regularly (once a week or less) **					
Neutral/reserved/opposing	64.68 (791)	73.53 (75)	60.25 (303)	37.1 (69)	79.63 (344)
Encouraging/supportive	**28.62 (350)**	**21.57 (22)**	**36.38 (183)**	**59.14 (110)**	**8.1 (35)**
PNA/do not know	6.7 (82)	4.9 (5)	3.38 (17)	3.76 (7)	12.27 (53)
If used daily **					
Neutral/reserved/opposing	83.24 (1018)	94.12 (96)	84.1 (423)	69.35 (129)	85.65 (370)
Encouraging/supportive	**10.55 (129)**	**2.94 (3)**	**12.33 (62)**	**29.03 (54)**	**2.31 (10)**
PNA/do not know	6.21 (76)	2.94 (3)	3.58 (18)	1.61 (3)	12.04 (52)
Attitudes towards cannabis use for medicinal purposes					
If only used once or twice **					
Neutral/reserved/opposing	19.71 (241)	33.33 (34)	12.33 (62)	8.6 (160)	29.86 (129)
Encouraging/supportive	**73.26 (896)**	**63.73 (65)**	**82.9 (417)**	**86.56 (161)**	**58.56 (253)**
PNA/do not know	7.03 (86)	2.94 (3)	4.77 (24)	4.84 (9)	11.57 (50)
If used once in a while or occasionally **					
Neutral/reserved/opposing	24.61 (301)	43.14 (44)	14.91 (75)	12.9 (24)	36.57 (158)
Encouraging/supportive	**66.15 (809)**	**50.98 (52)**	**78.13 (393)**	**81.72 (152)**	**49.07 (212)**
PNA/do not know	9.24 (113)	5.88 (6)	6.96 (35)	5.38 (10)	14.35 (62)
If used regularly (once a week or less) **					
Neutral/reserved/opposing	34.01 (416)	47.06 (48)	24.85 (125)	15.59 (29)	49.54 (214)
Encouraging/supportive	**56.75 (694)**	**45.1 (46)**	**68.59 (345)**	**79.57 (148)**	**35.88 (155)**
PNA/do not know	9.24 (113)	7.84 (8)	6.56 (33)	4.84 (9)	14.58 (63)
If used daily **					
Neutral/reserved/opposing	53.97 (660)	73.53 (75)	47.12 (237)	40.86 (76)	62.96 (272)
Encouraging/supportive	**37.45 (458)**	**20.59 (21)**	**46.72 (235)**	**55.38 (103)**	**22.92 (99)**
PNA/do not know	8.59 (105)	5.88 (6)	6.16 (31)	3.76 (7)	14.12 (61)

The numbers are in bold to highlight the encouraging behavior towards cannabis use and the change between types of users

^**^ *P*-value < 0.01

**Table 6:** Perceived harmfulness of cannabis by cannabis user types (*N* = 1223)

	Total (*N* = 1223)	Neither recreational nor medicinal (*N* = 102)	Only recreational (*N* = 503)	Both recreational and medicinal (*N* = 186)	PNA recreational, no medicinal (*N* = 432)
	%(*n*)	%(*n*)	%(*n*)	%(*n*)	%(*n*)
If tried only once or twice **					
Very harmful	5.4(66)	12.75(13)	0.8(4)	0.54(1)	11.11(48)
Harmful	4.58 (56)	10.78(11)	1.19(6)	0(0)	9.03(39)
Somewhat harmful	16.6(203)	14.71(15)	13.92(70)	12.9(24)	21.76(94)
Not harmful	**25.02(306)**	**26.47(27)**	**24.25(122)**	**23.66(44)**	**26.16(113)**
Not harmful at all	**39.66(485)**	**21.57(22)**	**56.86(286)**	**59.68(111)**	**15.28(66)**
PNA/do not know	8.75(107)	13.73(14)	2.98(15)	3.23(6)	16.67(72)
If used once in a while or occasionally**					
Very harmful	4.17(51)	9.8(10)	0.6(3)	1.08(2)	8.33(36)
Harmful	5.97(73)	12.75(13)	1.79(9)	1.08(2)	11.34(49)
Somewhat harmful	14.47(177)	20.59(21)	8.95(45)	4.84(9)	23.61(102)
Not harmful	**33.69(397)**	**29.41(30)**	**36.98(186)**	**32.8(61)**	**31.25(135)**
Not harmful at all	**32.46(397)**	**15.69(16)**	**48.11(242)**	**56.45(105)**	**7.87(34)**
PNA/do not know	9.24(113)	11.76(12)	3.58(18)	3.76(7)	17.59(76)
If used regularly (once a week or less)**					
Very harmful	8.99(110)	16.67(17)	1.79(9)	2.15(4)	18.52(80)
Harmful	14.55(178)	23.53(24)	8.75(44)	1.61(3)	24.77(107)
Somewhat harmful	23.96(293)	23.53(24)	27.04(136)	18.28(34)	22.92(99)
Not harmful	**26.66(326)**	**18.63(19)**	**34.19(172)**	**38.71(72)**	**14.58(63)**
Not harmful at all	**15.62(191)**	**4.9(5)**	**22.47(113)**	**33.87(63)**	**2.31(10)**
PNA/do not know	10.22(125)	12.75(13)	5.77(29)	5.38(10)	16.9(73)
If used daily **					
Very harmful	28.21(345)	45.1(46)	17.69(89)	9.68(18)	44.44(192)
Harmful	21.5(263)	20.59(21)	22.07(111)	15.05(28)	23.84(103)
Somewhat harmful	27.39(335)	18.63(19)	36.78(185)	44.09(82)	11.34(49)
Not harmful	**8.34(102)**	**2.94(3)**	**10.74(54)**	**17.2(32)**	**3.01(13)**
Not harmful at all	**4.25(52)**	**1.96(2)**	**5.77(29)**	**10.75(20)**	**0.23(1)**
PNA/do not know	10.3(126)	10.78(11)	6.96(35)	3.23(6)	17.13(74)

The numbers are highlighted to show the perception of cannabis use as being "not harmful" and how the perceptions change between types of users

^**^P-value < 0.01

**Table 7:** Knowledge and attitude towards legislative changes by cannabis user type (*N* = 1223)

	Total (*n* = 1223)	Neither recreational nor medicinal (*N* = 102)	Only recreational (N = 503)	Both recreational and medicinal (*N* = 186)	PNA recreational, no medicinal (*N* = 432)
	% (*n*)	% (*n*)	% (*n*)	% (*n*)	% (*n*)
The new medicinal cannabis law in Lebanon would: **					
Regulate the cultivation of cannabis among farmers… (Correct answer)	58.79 (719)	56.86 (58)	63.22 (318)	66.13 (123)	50.93 (220)
Non-correct answers	41.21 (304)	43.14 (44)	36.78 (185)	33.87 (63)	49.07 (212)
Legalization of cannabis in Lebanon for medicinal use only encourage trying other illegal drugs for recreational purposes					
Yes	4.42 (54)	1.96 (2)	4.17 (21)	4.3 (8)	5.32 (23)
No	86.59 (1059)	87.25 (89)	89.26 (449)	86.56 (161)	83.33 (360)
PNA/unsure	8.99 (110)	10.78 (11)	6.56 (33)	9.14 (17)	11.34 (49)
Legalization of cannabis in Lebanon for recreational use encourage trying other illegal drugs for recreational purposes **					
Yes	5.31 (65)	2.94 (3)	6.16 (31)	3.23 (6)	5.79 (25)
No	84.96 (1039)	88.24 (90)	85.29 (429)	87.1 (162)	82.87 (358)
PNA/unsure	9.73 (119)	8.82 (9)	8.54 (43)	9.67 (18)	11.34 (49)
Attitude towards legalization of cannabis in Lebanon for medicinal purposes **					
Neutral/reserved/opposing	19.38 (237)	27.45 (28)	8.55 (43)	3.23 (6)	37.04 (160)
Encouraging/supportive	74.41 (910)	67.65 (69)	87.67 (441)	95.16 (177)	51.62 (223)
PNA/do not know	6.21 (76)	4.9 (5)	3.78 (19)	1.61 (3)	11.34 (49)
Attitude towards legalization of cannabis in Lebanon for recreational purposes **					
Neutral/reserved/opposing	38.02 (465)	66.67 (68)	19.88 (100)	14.52 (27)	62.5 (270)
Encouraging/supportive	54.62 (668)	29.41 (30)	75.94 (382)	83.87 (156)	23.15 (100)
PNA/do not know	7.36 (90)	3.92 (4)	4.17 (21)	1.61 (3)	14.35(62)

^**^ *P*-value < 0.01

### Cannabis consumption by sociodemographics

As shown in Table 3, the older young adults (21–24), males, those employed at the time of the survey, living with non-family members, and who perceived themselves as being a little/lot richer than most were statistically significantly more present in the cannabis user subtypes than non-cannabis users (Table 3).

### Cannabis and other substances consumption

The prevalence of lifetime use of various substances was as follows: alcohol (80%), cocaine (11%), hallucinogens (10%), ecstasy/MDMA (12%), Salvia (12%), and any benzodiazepines (11%). The distribution of cannabis user types by each substance is shown in Table 4, but statistically significant differences were only investigated for any illicit substance use (other than cannabis), alcohol consumption, and any psychoactive prescription drug use. As can be seen, any illicit drug use (other than cannabis) was reported by 25% of the young adult sample but only 1% of the non-cannabis users, 36% of the recreational users only, and 58% of the recreational/medicinal users. Similarly, any prescription drug use was reported by 13% of the total young adult sample: 3% of the non-cannabis users, 16% of the recreational users only, and 28% of both recreational/medicinal users. Compared to recreational users only, the prevalence of drug use was higher in the dual motive users. For alcohol consumption, the trends were slightly different. First, the absolute difference between the percentage of lifetime users in the total sample and non-cannabis users is not that marked (79% vs. 54%, respectively) as in the case of illicit drug use and prescription drugs. Second, no differences were observed between recreational cannabis users only and dual motive users (95% and 92%, respectively) (Table 4).

### Attitudes towards cannabis use for recreational and medicinal purposes

A few observations can be made. First, regardless of cannabis use status, the percentage of those who were supportive/encouraging was markedly lower as the frequency of recreational use increased (Table 5). Second, the percentage of young adults pro cannabis use or supportive/encouraging of recreational use (whether once or twice, occasionally, weekly, or daily) was consistently higher for dual motive users than recreational users only (always lowest for the non-cannabis users) (Table 5). When recreational users only are compared to dual motive users, the absolute difference in the percentage supporting/encouraging recreational cannabis use is narrowest when it comes to “once or twice”, and consistently widens as frequency increases [prevalence ratio 1.13 for “once or twice”, 1.25 for “occasionally”, 1.64 for “regularly”, and 2.4 for “daily”] (Table 5). In other words, dual motive users were even more lax than recreational users only with increasing cannabis frequency. This trend was not as clearly observed when young adults were asked about medicinal cannabis use. The third observation was that, within each cannabis user type, a higher percentage was supportive/encouraging of medicinal than recreational use (e.g. within non-cannabis users, only 3% were supportive/encouraging of daily use of recreational cannabis vs. 21% for medicinal purpose) (Table 5).

### Perceived harmfulness of cannabis

As shown in Table 6, regardless of cannabis user status/type, the perception of harm increases with increased frequency of cannabis use. Still, for each cannabis frequency level, the percentage of perceived harmfulness was greatest in the non-cannabis users, followed by recreational users only, and least among dual motive users (i.e. recreational and medicinal users) (Table 6).

### Cannabis-related harms

Two-thirds of the lifetime cannabis recreational users only and three-quarter of the dual motive users reported using cannabis in combination with alcohol or other drugs so that their effects overlap. About half (51%) of recreational users only and two-thirds (68%) of dual motive users also reported operating a vehicle while under the influence of cannabis. Riding as a passenger in a motorized vehicle with someone who had used cannabis was least reported by the non-cannabis users (13%), followed by a significant majority of cannabis users (68% of the recreational users only and 77% of the dual motive users).

### Knowledge and attitudes towards legalization of cannabis

A substantial 40% of the young adult sample incorrectly answered the question related to the details of the new legislative change, lowest percentage of incorrect answers in the dual motive users (Table 7). As shown in Table 7, a very small percentage (2% of the non-cannabis users and about 4% of the cannabis users) believe that legalization of cannabis in Lebanon for medicinal use only would encourage trying other illegal drugs for recreational purposes. A higher percentage (though still small) felt the same way about legalization of recreational cannabis use (3% of non-cannabis users, 6% of recreational users only, and 3% of dual motive users). The majority was supportive of legalization of cannabis for medicinal use, lowest percentage among the non-cannabis users, followed by recreational users only and highest support among dual motive users (68%, 88%, and 95%, respectively) (Table 7). The support for legalization of cannabis for recreational use was much lower among the non-cannabis users (29%) and was comparably high among the cannabis lifetime users (76%-84%).

## Discussion

This first study following the historical passing of legislative change in Lebanon to legalize cannabis cultivation for medicinal use has highlighted and documented important differences between non-cannabis users, recreational users only, and dual motive users (both recreational and medicinal users). Our findings confirm that dual motive users are a subgroup that warrants further attention. In their study, Turna et al. (2020) found that compared to medical users, the dual motives group reported more daily cannabis use, used more alcohol and tobacco, and were more likely to use cannabis to treat psychiatric conditions [5]. In this paper, we note that even when compared to recreational users only, dual motive users have a riskier behavioural profile and a less conservative general attitude towards legalization and perception of cannabis-related harms. Worth noting that all the dual motive users in our study had first used cannabis recreationally (on average, 2 years before medicinal use) compared to about 75% of an Australian sample [17]. From a harm reduction perspective, this could mean that the “fear of diversion/conversion” from medical to recreational use is less relevant in the case of cannabis since most users begin to us cannabis for non-medical purposes. Indeed, the overwhelming majority of cannabis users reported “experimentation” as their main reason for initiating use. Nonetheless, despite their “benign motives”, all cannabis users in the study are in fact using cannabis “illegally” and are at risk of being prosecuted by law. Understanding patterns of use and motives could, therefore, inform national drug policies, including the Lebanese drug law, which has been revisited and is being reviewed by the Parliamentary Committee for Human Rights [18].

In the United States (US), trends of annual cannabis prevalence in college students have been fluctuating since the 1960s, reaching an all-time high (44%) in 2020 (Schulenberg et al., 2021). In Europe, cannabis remains the most widely consumed substance, with about 16% of young adults aged 15–34 reporting past-year use (ranging between 3 and 23% across different European countries) (EMCDDA, 2022). In Lebanon, though country-level data on cannabis use date back to 2003, [19], more recent university surveys have consistently found cannabis to be the most widely used illicit drug [20]. Besides impact on consumption, legalization of cannabis use, namely recreational, has been associated with more positive health perceptions of cannabis use [21]. Studies from Europe and North America have shown that the legalization of cannabis use for medicinal purposes did influence the perception of risk regarding cannabis [22]. Latest data from the US show that the percentage of young adults (19–30 years) residing in the US who reported marijuana use as “risky” has reached an all-time low (5–8%) in 2020 [23]. In addition, personal disapproval of cannabis use, whether experimental, occasional, or even regular is on a declining trend [23]. Though cannabis is an illegal substance in Lebanon, close to 55% of young adults are supportive of its legalization for recreational use and three-quarters for medicinal use. This is similar to the US estimates where voters supported the legalization of medicinal marijuana at significantly higher rates than recreational marijuana [24]. Overall, the support for some form of legalization is also in line with observations from the US [25], where attitudes have shifted in a liberal direction over time [26].

Studies have shown that about half of lifetime cannabis users proceed to use other illicit drugs [27, 28], which is similar to our findings, although we further highlight an important distinction between the prevalence of other illicit drug use in recreational users only (36%) versus dual motive users (58%). Other cannabis-related harms include a substantial proportion of cannabis users admitting to operating a vehicle within 2 h of cannabis consumption. The previous literature clearly shows how drug and/or alcohol combinations contribute to increased car crashes [29]. Recreational use (with or without concomitant medicinal use) can, therefore, be harmful beyond consumption per se, particularly when users get behind the wheel, highlighting the need for injury prevention programmes to reduce driving under the influence of cannabis regardless of its legal status [30]. The fact that the majority also admitted to intentionally combining cannabis with alcohol or drugs to heighten drug effects also warrants being addressed through harm reduction policies and programmes especially that a recent review concluded that the co-use of alcohol and cannabis is associated with more impairments, higher and more frequent consumption levels, and greater likelihood of comorbidity [31]. Use of cannabis has also extended beyond traditional private locations due to the spread of new methods of consumptions, through edibles and vaporizers, leading to potential for indirect harm [32].

The issue of early substance use, including cannabis, must be addressed within the context of social determinants of health, which recognizes the broader social context of marijuana use as per the socio-ecological model approach [33, 34]. In our study, the predominant majority (almost eight in 10 young adults) reported that they were in the company of a close friend when they first tried cannabis is an indication that young adults select peers with similar current marijuana consumption patterns, and/or adjust to their peers’ group behaviour over time [35].

Other important findings relate to the possibility of significant cannabis uptake following the multiple crises that Lebanon witnessed in 2020, similar to what was witnessed following the 9/11 incident in the US [36]. During the year preceding our online survey, Lebanon had experienced a series of crises starting with the ongoing financial crisis (October 2019), followed by the pandemic (February 2020), and ending with the landmark Beirut Port explosion (August 2020). Inevitably, the multiple crises have negatively affected the mental health of the population, both young people and adults [37, 38]. The link between this succession of adverse events and cannabis initiation cannot be ascertained from our cross-sectional survey. Still, a quarter of the lifetime recreational cannabis users, and 40% of the medicinal users used cannabis for the first time within the year prior to the survey date, and we know from other local data that increase in substance use initiation/frequency were reported [39].

From a health policy standpoint, the extent of self-medication with cannabis among the medicinal users is quite significant, with only 2% having been “prescribed” cannabis by a healthcare provider/pharmacist. While the fact that cannabis use remains illegal in the country and not formally available for prescribing, a prescription may have been issued by a physician from a country where such service is available, or young adults may have understood “prescribed” interchangeably with “recommended” and not an issuance of a formal prescription. Though not prescribed for the predominant majority, this fact does not exclude the possibility that cannabis may have been “recommended” for use, by a clinician or a pharmacist. This is of particular concern since most physicians, as per a recent systematic review, are unaware of the substance’s clinical benefits and adverse effects and hence are not in a position to provide advice [40].

The study has its limitations. Intrinsic to most online surveys is the risk of selection bias. Such an online sample not representing all residents of Lebanon within that age group. The study was conducted following the news of legalization cannabis cultivation for medicinal use, which coincided with the COVID-19 lockdowns; thus, an online survey was the only feasible method of data collection. The second limitation is the complexity in defining recreational vs. medicinal cannabis use. We defined medicinal use as “any consumption to cure, treat or prevent a physical or psychiatric disease” and recreational use as “any consumption for pleasure or to induce an alternate state of consciousness”. In the case of medicinal use, our definition did not distinguish between the use of THC- and CBD-based products. Overlap between these products is anyway well documented and quality control almost impossible [41]. The third limitation, though insightful, is the substantial percentage (36%) of young adults who chose not to disclose their cannabis use status, despite the fact that the survey was online, self-administered, and anonymous. Nonetheless, they completed the survey, and our analyses showed that their profile was similar to the non-cannabis users, which is most likely their cannabis use status. Therefore, their reluctance to report recreational cannabis consumption (and respond to the medicinal use question and all other survey questions) could be due to fear of being legally pursued, as safety and ethical concerns are not entirely resolved in online surveys despite researcher efforts to maintain privacy and integrity [42]. Further analyses showed that non-disclosers were more likely to be younger, of female gender, Lebanese, unemployed, living with parents or guardians, and not using any substances (an additional file shows this in more detail [see Additional file 1]). One solution moving forward is to employ different data collection methods. One study comparing findings from a traditional survey to indirect survey methods (the cross-wise model) found that the reported lifetime prevalence of cannabis use was two–three folds higher when using the indirect survey method, concluding that the latter may provide more accurate estimates keeping in mind both were administered online [43].

## Conclusion

Notwithstanding its limitations, the study from Lebanon, a developing country where cannabis is illegally cultivated, consumed and exported widely, offers an important snapshot of how youth consume and perceive cannabis, and how important differences do exist between different cannabis user types. The findings, though not representative of the overall young adult population, present opportunities of comparison with data and trends in other countries, where legislation has changed significantly, and continues to evolve as epidemiological research provides meaningful information on changes in attitudes and behaviour. Cannabis-related legislation is likely to continue to be decided on a local level and needs to rely on local understanding of the current state of affairs and the implications of any changes. The study clearly notes that legislative changes for medical cannabis cannot be undertaken in isolation given the complex overlap between medicinal and recreational use. Policy makers should be made aware of this reality and prepare for the wider consequences of their decisions. Drug control measures must be aligned with public health goals [44]. Therefore, local policies and public health programmes aimed at reducing the health, social, and economic costs of cannabis use in young adults must adopt a harm reduction approach. Harm reduction approaches could range from policy reform and legal changes to eliminate use or changing modes of cannabis use if the harm arises from drug use per se or decriminalization if the harms result from extensive policing and criminalization [45]. This is especially important given that successful legalization carries with it logistical challenges in the absence of a strong state apparatus (the case of Lebanon) able to guarantee trusted sources of production and safe distribution networks [14].

### Supplementary Information

**Additional file 1**: Lifetime use of cannabis for recreational purposes (*n*=1230)

## Data Availability

The datasets used and/or analyses during the current study are available from the corresponding author on reasonable request.

## Abbreviations

IRB: Institutional review board
LMIC: Low–middle-income countries
US: United States
