# Supplementary material for: Patterns of cannabis use, perception of harm, and perceived impact of legislative change in an online sample of young adults from Lebanon: insight on recreational users versus dual motive users
Source: Harm Reduct J. 2024 Feb 15;21:41. doi: 10.1186/s12954-024-00958-3 (PMC10868015; doi:10.1186/s12954-024-00958-3)
Supplement: Supplementary file 1 — Additional file 1: Lifetime use of cannabis for recreational purposes (n=1230) [file 12954_2024_958_MOESM1_ESM.docx]

## Lifetime use of cannabis for recreational purposes (n=1,230)^[[1]](#footnote-1)^

|  | **Lifetime Recreational cannabis Use** | |
| --- | --- | --- |
|  | **Prefer not to answer**  **(N=436)** | **Yes/No**  **(N=794)** |
| **Age of respondent**** | **%(n)** | **%(n)** |
| 18-20 | 38.76 (169) | 28.84 (229) |
| 21-24 | 61.24 (267) | 71.16 (565) |
| **Gender*** |  |  |
| Female | 57.31(247) | 31.07(243) |
| Male | 42.69(184) | 68.93(539) |
| Prefer not to answer | *(5)* | *(12)* |
| **Nationality*** |  |  |
| Dual Citizenship^[[2]](#footnote-2)^ | 99.5 (410) | 97.8 (751) |
| Non-Lebanese | 0.5(2) | 2.2(17) |
| Prefer not to answer | *(24)* | *(26)* |
| **Country of residence for most of their life** |  |  |
| Lebanon | 98.25(393) | 97.96(721) |
| Other | 1.75(7) | 2.04(15) |
| Prefer not to answer | *(36)* | *(58)* |
| **Student enrollment status*** |  |  |
| Not a student | 12.18(52) | 30.35(241) |
| Student full/part-time | 87.82(375) | 69.65(553) |
| Prefer not to answer | *(9)* | *(20)* |
| **Highest level of education** |  |  |
| Secondary school/high school | 24.88(103) | 20.66(157) |
| Some university education/not a completed degree | 22.46(93) | 21.97(167) |
| Bachelor’s Degree (BS/BA) | 43(178) | 45.26(344) |
| MSc. / MD / PhD | 9.66(40) | 12.11(92) |
| Prefer not to answer | *(15)* | *(19)* |
| **Job status at the time of the survey*** |  |  |
| Working | 20.85(88) | 38.34(296) |
| Not working | 79.15(334) | 61.66(476) |
| Prefer not to answer | *(14)* | *(22)* |
| **Living arrangement*** |  |  |
| With parents or guardians | 90.47(389) | 79.41(621) |
| Other | 9.53(41) | 20.59(161) |
| Prefer not to answer | *(6)* | *(12)* |
| **Perceived SES*** |  |  |
| Little/LOT poorer | 10.27(42) | 21.31(134) |
| About the same | 60.15(246) | 36.72(231) |
| Little/LOT richer | 29.58(121) | 41.9(264) |
| Prefer not to answer | *(27)* | *(21)* |
| **Lifetime alcohol*** |  |  |
| Yes | 61.16(263) | 89.72(707) |
| No | 38.84(167) | 10.28(81) |
| Prefer not to answer | *(6)* | *(6)* |
| **Lifetime illicit drugs use*** |  |  |
| Yes | 1.02(4) | 19.59(116) |
| No | 98.98(387) | 80.41(476) |
| Prefer not to answer | *(2)* | *(159)* |
| **Lifetime prescription drugs use*** |  |  |
| Yes | 5.28(21) | 17.39(133) |
| No | 94.72(377) | 82.61(632) |
| **Encouraging/supporting recreational cannabis** |  |  |
| If only used once or twice* | 14.22 (62) | 49.12 (390) |
| If used once in a while or occasionally* | 17.2 (75) | 61.34 (487) |
| If used regularly (once a week or less)* | 8.26 (36) | 39.8 (316) |
| Daily* | 2.29 (10) | 14.99 (119) |
| **Encouraging/supporting medicinal cannabis** |  |  |
| If only used once or twice* | 58.72(256) | 81.23(645) |
| If used once in a while or occasionally* | 49.08(214) | 75.44(599) |
| If used regularly (once a week or less)* | 36.01(157) | 68.14(541) |
| Daily* | 22.71(99) | 45.34(360) |
| **Encouraging/supporting legalization of cannabis** |  |  |
| Medicinal cannabis* | 52.06(227) | 86.78(689) |
| Recreational cannabis* | 23.62(103) | 71.66(569) |

1. * Significant with p-value<0.05

   ** Significant with p-value<0.001 [↑](#footnote-ref-1)
2. Lebanese and Lebanese citizens of another country [↑](#footnote-ref-2)
